# Annotations

**Published:** 1890-03-22

**Authors:** 


### Annotations.

**Barmaids and their Friends.**

The days of the cynic and the scorner appear to be passing away. Women of the upper class are finding time in the midst of a variety of social engagements to take thought for other women less fortunate than themselves, and upon whom destiny presses with a grievous weight that threatens to crush all womanhood out of them. A goodly number of ladies, among them Lady Clark, Mrs. Caine, Mrs. Carus Wilson, Mrs. Bevan, and Miss Kinnaird, met last week, at the house of Miss Morley, 47, Grosvenor-street, to consider the condition of barmaids and girls employed in restaurants, with a view to legislation on the subject. The meeting was of a widely representative character, there being present members of the Girls' Friendly Society, the Women's Union, the National Vigilance Association, the British Women's Temperance Association, and the Church of England Temperance Association. What have barmaids to complain of, it may be asked? If everything he true that is said of the barmaid's life, a quite sufficient ground for complaint is the fact that they are barmaids. Miss Gough, of the Young Women's Christian Association, related some facts at the meeting which she had learnt during her recent visits to the girls. These are samples of the facts: Many of the girls work a hundred hours a week; that is, more than sixteen and a half hours a day. During the greater part of this time they have to stand, and if possible to look amiable. For working a hundred hours a week the wages of numbers of the girls range from five to ten shillings. At the lower rate the wages amount to a little more than a halfpenny an hour. Of course they are fed, says the objector! No doubt, for if they were not fed they would be dead. No woman can live and work a hundred hours a week, and dress herself decently, on five shillings a week, or even on ten. The sanitary condition of the sleeping accommodation of many of the barmaids was said to be "disgraceful." But is not the whole history of their treatment, as thus disclosed, "disgraceful," and worse than "disgraceful"? The ladies, under Miss Morley's presidency, resolved to strive for legislation, and they were right. This is clearly not a question which can be dealt with by voluntary agency, though voluntary agency may do much to help legislation forward. The report of the meeting states that the rough draft of a Bill which it is intended to introduce into Parliament was read and warmly discussed. The "warmth" of the discussion is a good augury. If ladies discuss a question coldly all hope of good results may be promptly abandoned. The Bill provides that the hours of labour for barmaids shall be limited to seventy-two a week; that restaurant and public-house premises shall be kept in a wholesome sanitary condition; and that inspectors shall be appointed to periodically examine and report upon them. These provisions are very good so far as they go; but another resolution which the meeting passed, and which strongly deprecated the employment of women in bars and drinking-places at all, commends itself more to our judgment. In America it is the custom to employ men waiters in drinking-places. It would be much better if the same practice prevailed in this country. Women were never intended to face the coarse, vulgar, and often brutal ordeal of a drinking bar.

**Inebriate Women and Girls.**

Mrs. Temple, the wife of the Bishop of London, presided last week at a morning concert given at St. James's Hall in aid of the Homes and Shelters for inebriate women and girls. That women by nature timid, gentle, and trusting, should come in course of years to drink strong drink, and to love it, to make themselves brutish foul, unclean, and drink-mad—this is one of the most pitiable accompaniments of our modern life and history. That women, we say, should do this is inexpressibly saddening and tragic; but that girls, sometimes mere children, who ought to be pure, and hardly even to know of the existence of drink and drunkenness—that these should find their way into the horrible pit and miry clay of baseness, animalism, and drink-madness—this is a thing that no words can express the pity of. Side by side with these terrible growths of modern city life there are developing agencies full of heroic Christian virtue. Christianity, unlike ancient and even modern philosophy, believes with a sublime confidence that it has got down to the bed-rock of eternal verity in morals. Vice, it says, is bad; illicit passion is bad; self-indulgence is bad; everything that is opposed to pureness of heart and life, to truthfulness, to due regard for the claims of others—all these are bad: they are not merely inartistic, or unamiable; but they are positively, certainly, scientifically, and everlastingly vicious and bad. On the other hand, absolute purity and virtue are right, obligatory, imperative; they are not merely pleasant, beautiful, and desirable. Armed with these weapons of scientific precision, of the keenest temper, and of the most enduring quality, Christian men and women first subdue their own spiritual enemies and then go down into the arena of moral strife to fight with all possible ardour and persistence. Mrs. Temple has set herself to do what she can to lift up those of her own sex who have fallen down by the way, worse than dead. That lady probably needs no inspiration from us; but she certainly will need the sympathy of her fellow-women and the practical help of both women and men. If an agency for the recovery of inebriate women and girls, of sufficient extent and energy to cover the ground that needs to be covered, could be established; and if it could be furnished with resources sufficient for its needs, a work might be done which could not be surpassed in importance by any work known to benevolence. Women of ample leisure and of liberal means abound on all hands. It cannot but be that many such women, having human hearts, find a life of fashion and self-indulgence infinitely wearisome and stale. What a new life is possible for them if they could but be brought to enter upon it. Mrs. Temple will surely find many sisters eager to help in her newly-found work. If it should be otherwise, then it will show that English women are made of the stuff that a certain historical pharisee was made of; and it may prove to be more tolerable in the day of judgment for inebriate women and girls than for them.

**A Modern Mountebank.**

The *Hospital Gazette* of February 22nd contains a note headed "Sequah in the Provinces." It is stated that his treatment is a form of somewhat violent massage, a procedure which might be more often adopted by regular practitioners. "Bounce," it is said, is the most important stock in trade of a quack doctor, and it is certainly one that the travelling quack Sequah possesses to a marvellous extent. Most quack doctors are admirable talkers. The more astounding their statements, the more they are believed in, provided they are uttered with sufficient appearance of confidence. Among other things, Sequah is a marvellously rapid extractor of teeth, an operation which he performs accompanied by a brass band. Now the *Hospital Gazette* is rather late in the day. In The Hospital of October 12th last, under the heading "A Phase of Modern Medicine," we gave a full account of Sequah and his doings. There is something, however, to be learnt, even from a man like Sequah. What he does know he evidently makes the most of, and the single operation he performs he performs excellently well. The ordinary practitioner would be none the worse if with his greatly superior knowledge and resources he would strive to bring that knowledge more completely and constantly to the service of every individual patient. A licence to practise sometimes means a licence to spend life both lazily and incompetently.

